# *Nanoscale Advances*, a new gold open access journal joins the nanoscale journal family

**DOI:** 10.1039/c8na90004c

**Published:** 2019-01-08

## Abstract

The editorial team introduce the Royal Society of Chemistry’s newest nanoscience and nanotechnology journal, *Nanoscale Advances*.
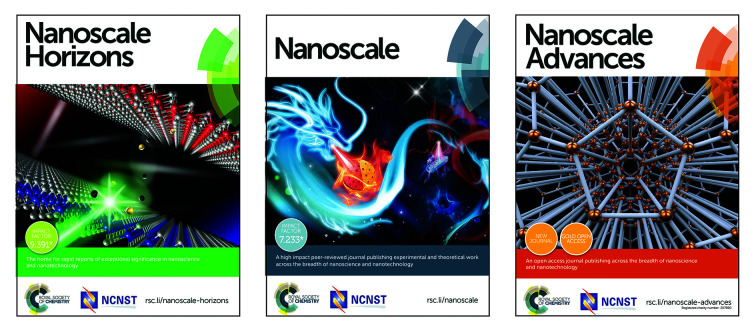

Welcome to the inaugural issue of *Nanoscale Advances*, the newest member of the nanoscale journal family published by the Royal Society of Chemistry. *Nanoscale Advances* is an international gold open access journal publishing across the breadth of nanoscience and nanotechnology. Our focus is on quality, reproducible new work that makes an important advance to the existing literature, as well as making it accessible to a global, interdisciplinary network of nanoscale researchers through gold open access publication.

All articles published are free to read on the *Nanoscale Advances* website and article-processing charges are currently waived, so it is free for authors to publish until mid-2021.

## Launch

At the Royal Society of Chemistry, we want to make sure that our journal portfolio represents the needs of our community. The publishing landscape as a whole is rapidly changing and we are committed to supporting long term, sustainable open access models. *Nanoscale Advances* is our first journal that will be gold open access from launch, and we chose a name that builds on the established open access brand of *RSC Advances*. We hope that, with this launch, we can better serve our community who want to publish high impact, open access, and nanoscience-targeted research. For more information on our open access policies, visit our website.

## The nanoscale journal family

Science and technology require collaboration to thrive and in this spirit, *Nanoscale Advances* joins the nanoscale journal family, *Nanoscale Horizons* and *Nanoscale*, as a collaborative venture between the Royal Society of Chemistry and a leading nanoscience institute: the National Center for Nanoscience and Technology (NCNST) in Beijing, China.

**“In the light of current trends, the Royal Society of Chemistry has made a significant step towards realizing the perfect “nanoscience” synergy by launching *Nanoscale Horizons*, *Nanoscale*, and *Nanoscale Advances*” – Professor Dirk Guldi, Editor-in-chief** 
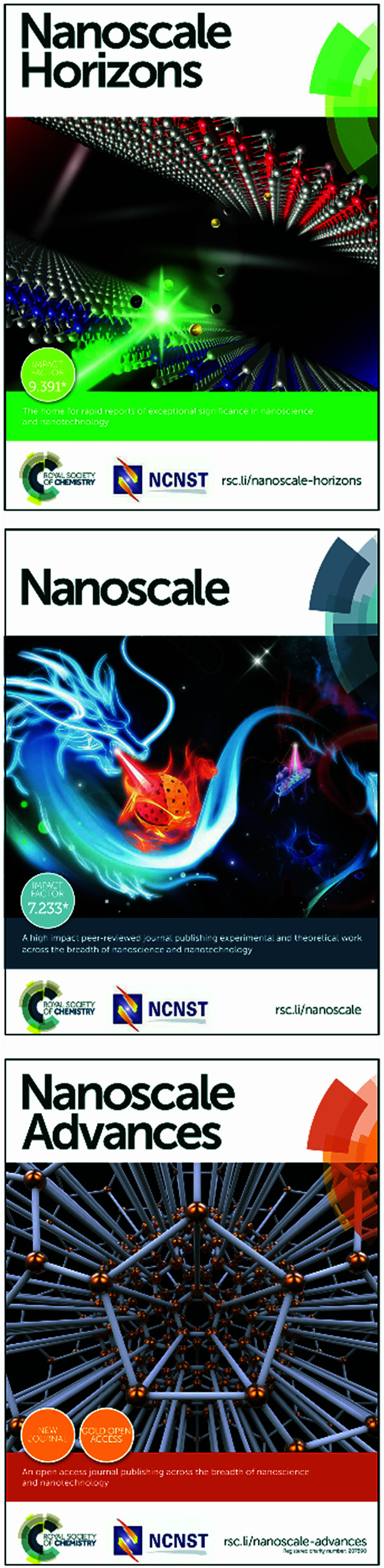


*Nanoscale* (impact factor 7.233) has cemented its position over the last ten years as the largest high impact nanoscience journal, publishing high quality work across the interdisciplinary field of nanoscience and nanotechnology. In 2016, *Nanoscale Horizons* was launched as a sister journal to capture truly cutting-edge ideas and new concepts in the field. With an inaugural partial impact factor of 9.391, it publishes exceptionally significant work of interest to the broad nanoscience community. As the third member of this nanoscale journal family, *Nanoscale Advances* will complement our existing journals as a venue for quality and important work that advances the existing literature.

## *Nanoscale* and *Nanoscale Advances* cooperation

All three journals in the nanoscale journal family share an identical subject scope, which means that we can offer an efficient manuscript transfer service, where appropriate. Our hope is that, no matter which journal you submit to, we will find the most appropriate home for your work with the minimum hassle for you.

To facilitate this, *Nanoscale* and *Nanoscale Advances* have a shared Editorial Board, so the same high profile Associate Editors handle manuscripts for both journals. This shared responsibility ensures that there is a common overview and a consistent application of expertise, streamlining the assessment process for our authors and our reviewers. If you submit to *Nanoscale* and are offered a transfer to *Nanoscale Advances*, the same Associate Editor will handle your article in both journals. This reduces possible delays and ensures an efficient transfer of the history of your article, including any reviewer comments. In addition, several of our Associate Editors also act as Scientific Editors on *Nanoscale Horizons*, helping ensure articles meet the stringent criteria required for publication.

The Editorial Board is led by two exceptional researchers, both leading figures in the nanoscience community.
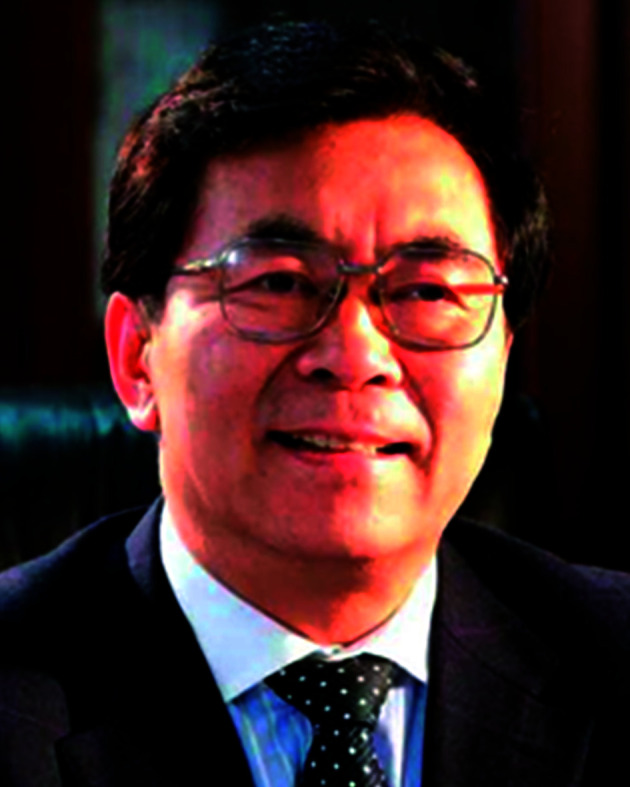


**Editor-in-chief Professor Chunli Bai** is Director of the NCNST, as well as the President of the Chinese Academy of Sciences. As a prominent figure, both in Asia and internationally, he provides substantial experience and prestige to the journal.
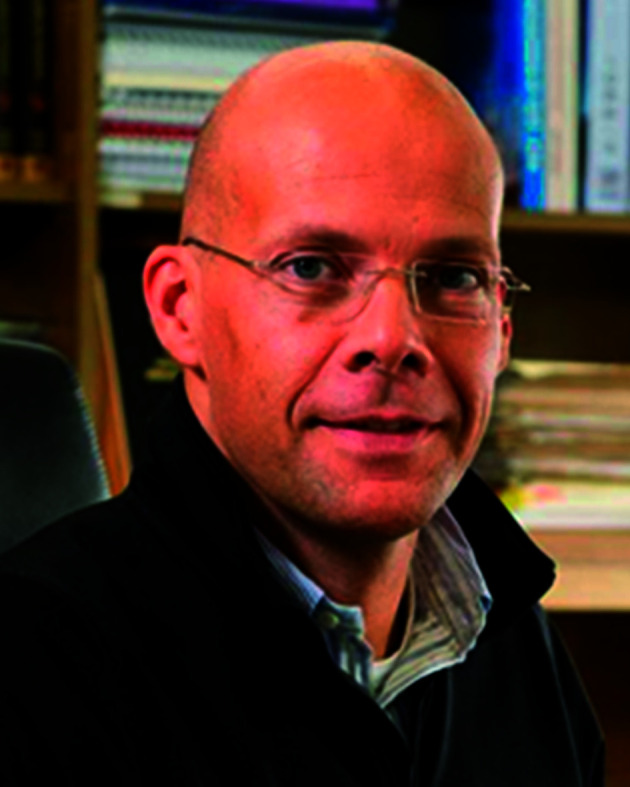


**Editor-in-chief Professor Dirk Guldi** also occupies a Scientific Editor position on the Editorial Board of *Nanoscale Horizons*. Working across all three journals, he is perfectly placed to ensure close ties between the members of the nanoscale family.

## Moving forward

We hope that you enjoy reading our first issue and that you will join us in some of our upcoming initiatives! Keep an eye out for future virtual themed collections featuring articles published in our nanoscale journals.

Follow us on Twitter, Facebook, or sign up to our newsletter to keep up to date with the latest news and some of our most recently published content.

Alternatively, come and meet us! Members of the team will be attending the Spring MRS in Phoenix, USA in April 2019, and we will be running a symposium during ChinaNANO at the NCNST in China in late summer 2019.

Finally, we are committed to developing and taking *Nanoscale Advances* forward so that the journal fully meets the needs of our authors and readers. We always welcome comments, suggestions, and feedback, so please do contact us at nanoscaleadvances-rsc@rsc.org with your views and feedback.

Professor Dirk Guldi, Editor-in-chief

Dr Sam Keltie, Executive Editor

Dr Michaela Mühlberg, Deputy Editor

Dr Hannah Kerr, Development Editor

## Supplementary Material

